# Annotations

**Published:** 1891-05-02

**Authors:** 


### Annotations.

**Rational Dress.**

The words "Rational Dress" connote women. Nobody thinks of reasoning with men on the subject; it is taken for granted as a matter of course that they dress rationally. For our part, so long as the tall hat remains the chief symbol of the dignity of the British man, we shall hold that he has little reason to congratulate himself on his superior rationality. The divided skirt seems, to some, to represent the perfection of rationality, and the undivided skirt to be the expression of all that is inconsequent and stupid. But why is the divided skirt more rational than the undivided for women? This is a question which was not discussed by Carlyle in his Clothes Philosophy. Two questions strike down to the root of the matter; and these are concerned with the reasons which should direct the shape of women's dress. The woman, like the man, dresses for protection against the weather and for beauty. It will be admitted by every doctor that in our climate, at any rate, protection against the weather is fundamentally the more important. But in this respect the ordinary dress of women during the last few years has left little to be desired. The chest, the shoulders, the neck, the arms, the body, and the legs have all been well and warmly clad. The one question, therefore, that remains for discussion is that of beauty. Is the divided skirt more graceful, more elegant, more conducive to womanly dignity than the undivided? If we speak philosophically we are compelled to admit that it is entirely a matter of taste; but if we are called upon for a vote, our suffrages will be cast for the undivided skirt. The women of our own country, with whom of course we are most familiar, have established so secure a claim upon the respect and admiration of men by all their ways and doings, including the wearing of the undivided skirt, that we feel we should experience a very sharp pang if they were all suddenly to assume a different outward form and shape from that we are accustomed to. They would seem to be different women, and we do not want them to be different women; we are proud and happy for them to be the same women. But this is not logic, cries the advanced philosopher! "Hang logic," as Vicar Rogers said about theology. It is often better to be pleased and contented than to be logical. In any case we present our readers with our feelings on the subject, be they logical or be they illogical.

**The Royal Free asks for £20,000.**

The Royal Free Hospital in Gray's Inn Road asks for £20,000 wherewith to enlarge and improve its buildings. There are many general reasons why the Royal Free should obtain the money it asks for. Are there any reasons which are special and peculiar to this hospital? There are! The Royal Free issues what may be called a declaration of hospital principles: it publishes a confession of charitable faith. Here is a portion of the declaration: "Foreigners, strangers, and others, in sickness or disease, having neither friends nor homes, are admitted to the wards of this hospital on their own application, so far as the means of the charity will permit." All practical philanthropists will agree, and indeed all other persons who think about the matter, that there ought to be at least one hospital in London conducted on these peculiar lines, and that that hospital should be placed in a central and prominent position, and so distinctive in its external features as that every "foreigner," "stranger," and other sick person, "having neither home nor friends," may be able to find a temporary shelter and home without delay. If anything could excuse "sky signs" it would be such an institution as the Royal Free Hospital. One would almost like to see an immense sky sign towering up to the height of the dome of St. Paul's, and on it this truly divine and hope-inspiring inscription: "Royal Free Hospital: Open to foreigners, strangers, and the homeless and friendless sick on their own application." It is true that the hospital is obliged to add the words—"so far as the means of the charity will permit." But we want one hospital endowed with the means of receiving, at least for a time, every sick foreigner and homeless person who chooses to apply, and nowhere could such a hospital be more appropriately or centrally placed than where the Royal Free now stands in the Gray's Inn Road. It seems to us that the Royal Free owes it to its noble traditions to make a great effort to completely realise its own ideal in the wide and cosmopolitan sense we have here set forth; and it seems still more to be demanded of the people of London that they shall have and maintain liberally at least one central hospital which shall be free and open to all the world's sick and needy, at any rate for immediate shelter and treatment, on their own application alone. These are our suggestions for making the Royal Free ideally perfect and complete. In the meantime what is urgently wanted is the sum of £20,000 for rebuilding, and the bringing up of the hospital to the best sanitary standard. The Earl of Lathom has written a letter specially inviting generous persons to the festival dinner which is to be held next Saturday, May 2, at the Hotel Metropole. Lord Lathom is to preside, and among the stewards are Cardinal Manning, the Marquis of Dufferin, the Right Honourable Lord Hannen, Sir Edward Clarke, Q.C., M.P., the Right Honourable James Stansfeld, M.P., Sir J. Whittaker Ellis, Bart., M.P., Mr. Alfred De Rothschild, Mr. Sebag Montefiore, Dr. Crawford Hayes, F.R.C.P., Dr. Samuel West, F.R.C.P., and many other thoroughly judicious and trusted philanthropists. We are very anxious that this septennial festival shall fully justify the strenuous efforts put forth by the managers of the Royal Free Hospital and their many friends. We hope they will raise the £20,000 needed on the day of the dinner; but if this hope be not quite realised, we venture to ask all our readers to take at least a small share of the noble duty of providing shelter and medical care for foreigners, strangers, and other homeless sick. Those who are not able to attend the dinner may and we trust, will, send a large or a small subscription for the hospital building fund, before the end of next week; in order that the festival may realise what the managers hope for, and so enable them to do promptly the unique and truly charitable work for which their hospital was more especially founded.

**Gifts and Gratitude.**

We publish elsewhere a letter from the Chairman of the Governors' Board of the North-West London Hospital. The hospital, it may be remembered, was some time ago in financial straits. So urgent were the necessities of the moment that it was deemed advisable to issue a special appeal asking for £2,000 for current expenses. It was stated in the appeal that unless something like £2,000 could be provided, one of the principal wards in the hospital would have to be closed. The Committee under these circumstances bethought them of journalistic influence as a resource, and sent a communication to the office of The Hospital, asking us to call the attention of our readers to the threatened breakdown in their operations. It so happened that a member of the staff of The Hospital was very familiar with the north western district, and with its need for charitable medical relief. He wrote a plain statement of the facts of the case, and urged upon those who held philanthropy to be a duty the serious consideration of the necessities of the north-western districts. Within a few weeks of the appeal, and in consequence of the article in The Hospital, £1,100 were promised or received by the Treasurer. Baron Hirsch sent a cheque for £1,000, and Mr. John Maple a conditional offer of £100, the donor in each case stating that the gifts had been forwarded as the result of reading the article in The Hospital. We wish to point out to secretaries and managers that as The Hospital is now generally regarded as the representative organ of the medical charities, it can often render them very great service, provided always that the editor is supplied with facts and with the means of satisfying himself as to the merits of each particular case. So large, useful, and influential a work as is carried on by the medical charities was at a very great disadvantage whilst it remained without a special organ of its own to speak to the public in the language of truth and soberness. It has been our steadfast aim always to stand firm for the hospitals. Those institutions have for the most part cordially recognised our intentions and given us their loyal co-operation. Our task has thus been rendered comparatively easy. But we feel that if the managers and secretaries of hospitals would give themselves a little more trouble in keeping us thoroughly up to date in the details of their life and work we could render them infinitely more service than even we have rendered them in the past. We commend to those managers and secretaries the consideration of the letter of the Treasurer of the North-West London Hospital and of the possibilities that are thereby revealed.

